# Non-psychotic Outcomes in Young People at Ultra-High Risk of Developing a Psychotic Disorder: A Long-Term Follow-up Study

**DOI:** 10.1093/schbul/sbae005

**Published:** 2024-02-16

**Authors:** Anneliese E Spiteri-Staines, Alison R Yung, Ashleigh Lin, Jessica A Hartmann, Paul Amminger, Patrick D McGorry, Andrew Thompson, Stephen J Wood, Barnaby Nelson

**Affiliations:** Orygen, 35 Poplar Road, Parkville 3052, Victoria, Australia; The Centre for Youth Mental Health, University of Melbourne, Parkville 3052, Australia; Department of Social Work, Melbourne School of Health Sciences, University of Melbourne, 161 Barry St, Carlton 3053, Australia; Orygen, 35 Poplar Road, Parkville 3052, Victoria, Australia; The Centre for Youth Mental Health, University of Melbourne, Parkville 3052, Australia; Institute of Mental and Physical Health and Clinical Translation (IMPACT), Deakin University, Geelong, VIC 3320, Australia; School of Population and Global Health, University of Western Australia, Nedlands, WA 6009, Australia; Orygen, 35 Poplar Road, Parkville 3052, Victoria, Australia; The Centre for Youth Mental Health, University of Melbourne, Parkville 3052, Australia; Orygen, 35 Poplar Road, Parkville 3052, Victoria, Australia; The Centre for Youth Mental Health, University of Melbourne, Parkville 3052, Australia; Orygen, 35 Poplar Road, Parkville 3052, Victoria, Australia; The Centre for Youth Mental Health, University of Melbourne, Parkville 3052, Australia; Orygen, 35 Poplar Road, Parkville 3052, Victoria, Australia; The Centre for Youth Mental Health, University of Melbourne, Parkville 3052, Australia; Division of Mental Health and Wellbeing, Warwick Medical School, University of Warwick, UK; Orygen, 35 Poplar Road, Parkville 3052, Victoria, Australia; The Centre for Youth Mental Health, University of Melbourne, Parkville 3052, Australia; School of Psychology, University of Birmingham, Edgbaston, UK; Orygen, 35 Poplar Road, Parkville 3052, Victoria, Australia; The Centre for Youth Mental Health, University of Melbourne, Parkville 3052, Australia

**Keywords:** ultra-high risk for psychosis, UHR, comorbid mental disorders, long-term outcomes

## Abstract

**Background:**

The majority of individuals at ultra-high risk (UHR) for psychosis do not transition to a full threshold psychotic disorder. It is therefore important to understand their longer-term clinical and functional outcomes, particularly given the high prevalence of comorbid mental disorders in this population at baseline.

**Aims:**

This study investigated the prevalence of non-psychotic disorders in the UHR population at entry and long-term follow-up and their association with functional outcomes. Persistence of UHR status was also investigated.

**Study design:**

The sample comprised 102 UHR young people from the Personal Assessment and Crisis Evaluation (PACE) Clinic who had not transitioned to psychosis by long-term follow-up (mean = 8.8 years, range = 6.8–12.1 years since baseline).

**Results:**

Eighty-eight percent of participants at baseline were diagnosed with at least one mental disorder, the majority of which were mood disorders (78%), anxiety disorders (35%), and substance use disorders (SUDs) (18%). This pattern of disorder prevalence continued at follow-up, though prevalence was reduced, with 52% not meeting criteria for current non-psychotic mental disorder. However, 35% of participants developed a new non-psychotic mental disorder by follow-up. Presence of a continuous non-psychotic mental disorder was associated with poorer functional outcomes at follow-up. 28% of participants still met UHR criteria at follow-up.

**Conclusions:**

The study adds to the evidence base that a substantial proportion of UHR individuals who do not transition to psychosis experience persistent attenuated psychotic symptoms and persistent and incident non-psychotic disorders over the long term. Long-term treatment and re-entry into services is indicated.

## Introduction

Early intervention during the prodrome of psychosis has been shown to result in better outcomes for some people, including attenuation of psychotic symptoms, delay or even prevention of the onset of psychosis.^1,2^ The Ultra-High Risk (UHR) for psychosis criteria^3^ comprise trait and state risk factors to identify young people (15–30 years) at high risk of developing a psychotic disorder and enable early intervention. “High-risk” status is associated with transition to psychosis rates of 18% at 6 months, increasing each year up to 36% over 3 years.^4^

However, the majority of individuals do not transition to psychosis and the rate of transition in UHR samples has been declining.^3,5,6^ Given that the majority of individuals identified as UHR do not transition to psychosis, it is important to consider the range of other outcomes for these people.

Compared with healthy controls, UHR individuals have been shown to experience significantly lower quality of life and poorer functional outcomes, even if they do not transition to psychosis.^7–13^ Rates of comorbid psychiatric disorders as high as 80% have been observed in the UHR population^14,15^ and there is some evidence indicating that they report greater concern for their depressive and anxiety symptoms than their attenuated psychotic symptoms (APS).^10,16,17^

There has been growing interest in comorbid non-psychotic mental disorders in the UHR population, due to their high prevalence in this group.^9,14,15,18–20^ A systematic review identified 10 papers that investigated long-term follow-up of UHR cases who did not transition to psychosis, indicating that research on long-term clinical and functional outcomes in this population remains scarce.^9^ This review found that 20%–82% of these cases still met criteria for at least one clinical diagnosis at follow-up. However, only 4 of the 10 papers included in the review presented findings on outcomes beyond diagnosis of a DSM disorder.^2,19,21,22^ Lin et al^14^ reported 28% of the non-transitioning UHR sample experienced APS at follow-up, and this sub-group had a higher prevalence of comorbid non-psychotic mental disorders than the total sample. Onset of a new disorder occurred in 20% of the sample and continuous disorder in 52%.

These studies illustrate the importance of understanding the role of non-psychotic mental disorders in the UHR population, as these disorders may persist long-term. There is increasing acceptance in the field that treatments in the UHR stage should not focus solely on prevention of transition to psychosis but address the variety of outcomes and continuing clinical symptoms.^23,24,25^ However, other studies investigating long-term non-transitioned clinical outcomes have been either cross-sectional^26^ or did not have a follow-up period of greater than 2–3 years.^12,15,18,20,22^ One study^2^ had a longer follow-up period of 6 years but a small sample size (*n* = 44).

Studies examining the longer-term functional outcomes of non-transitioned UHR individuals have also had follow-up periods of less than 3 years.^8,9,11,21–24,27^ One of the challenges has been to establish long-term outcome on a sufficiently large sample. The current study aimed to further understand the long-term trajectory of non-psychotic disorders and UHR status in UHR individuals, and the relationship between non-psychotic disorders and functional outcomes. This may inform treatment options and facilitate management of mental illness in the UHR population.

The study investigated the long-term outcomes of mental disorders in a cohort of participants previously identified as UHR for psychosis at the Personal Assessment and Crisis Evaluation (PACE) clinic^28^—specifically, the prevalence of these disorders at entry and long-term follow-up and their relationship to functional outcomes over a 6–12 year follow-up period. This study builds upon Lin et al’s^14^ research on comorbid disorders in the UHR population by reporting on an extended follow-up period of a subset of the participants from that study to better understand their long-term clinical and functional outcomes.

Based on previous studies,^9,14,19^ we hypothesized: (a) high prevalence of non-psychotic disorders at both baseline and follow-up; (b) high rates of continuous and (c) incident non-psychotic disorders; (d) persistence of UHR status in a third of the sample. Due to a paucity of research on functional outcomes and their link to course of non-psychotic disorder, this was approached as an exploratory question.

## Methods

**Fig. 1.**
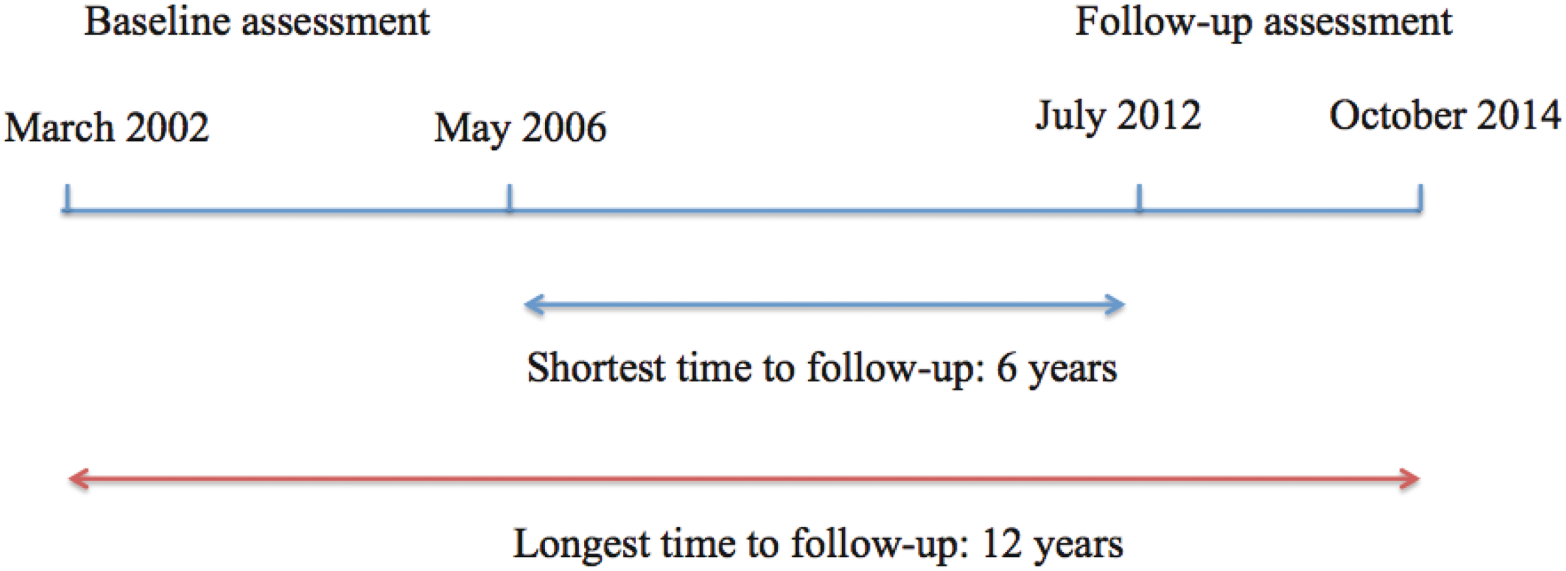
Recruitment timeline for follow-up assessment.

Individuals recruited for this study were previous patients at the PACE Clinic. PACE is a specialist clinical program for UHR patients aged 15–30 years at Orygen Youth Health in Melbourne. The current study re-assessed previously recruited research participants from PACE, with the follow-up conducted between 2012 and 2014, covering a period of 6–12 years since baseline assessment (figure 1). The current sample is a subset of the sample previously reported in Lin et al.^14^ The Lin et al^14^ sample had baseline assessments performed between 1993 and 2006. The current sample consists of the subset who had baseline assessments conducted between 2002 and 2006. Ethics approval was received from the Behavioural and Psychiatric Research and Ethics Committees of the North Western Mental Health Program.

### Participants

Participants were aged 15–30 years at baseline. They were eligible for the study if they met at least one of the three operationally defined UHR groups^1^ at baseline: (1) vulnerability group (schizotypal personality disorder or history of psychotic illness in a first degree relative) and decline in functioning or chronic low functioning; (2) APS, and/or (3) brief limited intermittent psychotic symptoms (BLIPS). Exclusion criteria were a previous psychotic episode (treated or untreated), organic cause for presentation, intellectual disability, and past antipsychotic exposure equivalent to a total lifetime haloperidol dose of >50 mg.

**Fig. 2.**
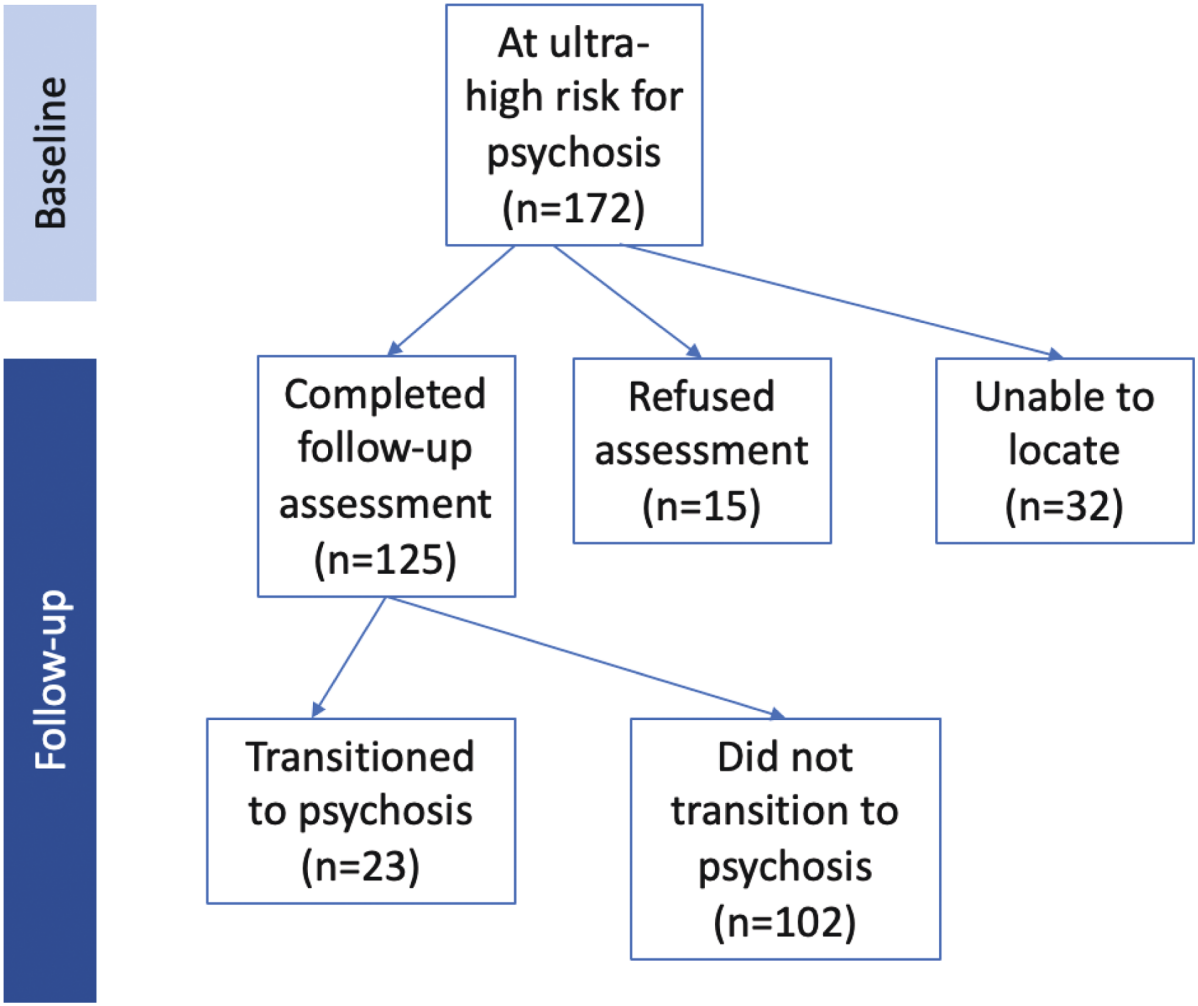
Composition of the sample of youth at ultra-high risk for psychosis.

Follow-up data was collected on a sample of 102 non-transitioning participants. 81 completed a face-to-face interview and 21 a telephone interview. Of these, 63 (62%) were female, 39 (38%) were male, a similar proportion to baseline (females *n* = 97, 56%, males *n* = 75, 42%). Gender was not related to whether a participant presented for an interview at follow-up, (χ^2^ (1, *n* = 102) = 1.046, *P* = .31). The average age of participants at follow-up was 27.0 years (ranging from 22 to 34 years, *SD* = 2.9 years). See figure 2 for sample composition.

### Measures

Ultra-High Risk for Psychosis status was determined using the Comprehensive Assessment of At-Risk Mental States (CAARMS), developed to assess the intensity, frequency, and duration of APS.^29^ Mental disorder diagnosis was assessed using the Structured Clinical Interview for DSM-IV.^30^ Transition to psychosis was determined using both the CAARMS and DSM-IV. Social and occupational functioning was assessed using the Social and Occupational Functioning Assessment Scale (SOFAS),^31^ which rates functioning independently of psychiatric symptoms. In the previous wave of follow-up of this sample,^14^ the GAF^32^ was used to assess functioning, which fails to separate social and occupational functioning from clinical symptomatology.

### Procedure

All participants had given informed consent for long-term follow-up at the time of their baseline interview (2002–2006). This group of participants was a subset (*n* = 172) of those followed-up in the Lin et al^14^ study (*n* = 226), the current study providing a longer follow-up period (a minimum follow-up period of 6 years on all participants, compared to the 2 years in the Lin et al^14^ study). A previously developed tracking approach^33^ was used to locate participants. Participants were invited to complete a face-to-face interview (full clinical assessment), with a telephone interview being conducted for those who declined face-to-face assessment (see Nelson et al^34^ for details). Participants who presented face-to-face provided written consent prior to their interview, verbal consent was given for telephone interviews.

### Statistical Analyses

The course of non-psychotic mental disorders was explored for individuals who had completed diagnostic assessments at both baseline and follow-up (current disorder or disorder present since baseline assessment). Those who did not receive a diagnosis of any DSM-IV disorder at either baseline or follow-up, or the time in between, were classified as *never*. For those who were diagnosed with a mental disorder at either baseline or follow-up, the course of their disorders was examined for three disorder groups: Individual disorders were grouped into mood (diagnosis of a major depressive disorder or non-bipolar disorder), anxiety (comprising panic disorder, GAD, social phobia, specific phobia, OCD, anxiety NOS, and PTSD) and SUDs as these were the most prevalent reported and the trajectories of these were disorders tracked over time. Participant disorders were classified as: *remitted* if the non-psychotic mental disorder present at baseline was not present at follow-up; *continuous* described participants whose non-psychotic mental disorder was present at both baseline and follow-up; participants who developed a non-psychotic mental disorder by follow-up that was not present at baseline were classified as having an *incident disorder*.^14^

Demographic variables were analysed using chi-square tests for independence (including gender, presence of mental disorders [at baseline and follow-up], and type of participation at follow-up [face-to-face/telephone]). Independent samples *t*-tests were used to analyse differences in age of participants. To investigate functional outcomes, one-way between-groups analysis of variance was used to investigate the relationship across the trajectories (remitted, continuous, incident, and never) and functional outcomes as measured by SOFAS.^31^ Multinomial regression was used to investigate the association between age, time to follow-up and diagnostic outcome. The likelihood of particular outcomes was determined using *P*-values <.05 and 95% confidence intervals (CI).

## Results

Presence of a mental disorder diagnosis [χ^2^ (3, *n* = 142) = 1.735, *P* = .629] or functioning score [Wald χ^2^ (1, *n* = 102) = 0.602, *P* = .438] at baseline was not related to whether participants were followed up or not. The majority of participants (*n* = 80, 78%) were born in Australia and spoke English as their main language (*n* = 83, 81.4%) with 17.6% (*n* = 18) of participants having both parents born overseas, and another 18.6% (*n* = 19) having one parent born overseas.

### Type and Prevalence of Mental Disorders at Baseline and Follow-up

**Fig. 3.**
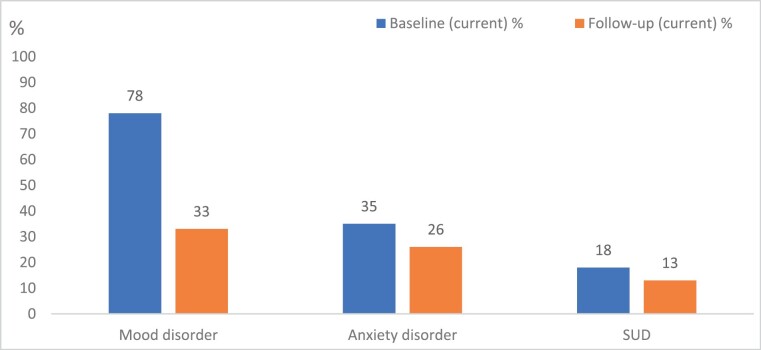
Prevalence of mental disorders at baseline and follow-up—major groups.

Mental disorder diagnoses were established at baseline if the participant had presented with symptoms of the disorder within a month prior to assessment. Only 12 participants (12%) did not meet criteria for a DSM-IV disorder at baseline. Mood disorder (A participant met criteria for mood disorder if they were diagnosed with any major depressive disorder or non-psychotic bipolar disorder) was the major diagnostic group for participants at baseline (*n* = 78, 78%) (figure 3). When grouped together, anxiety disorders were the next most common set of disorders (*n* = 35, 35%), followed by SUD (*n* = 18, 18%). (Note: a participant was classified as having an anxiety disorder if they had Generalized Anxiety Disorder, Social Phobia, specific phobia, Obsessive Compulsive Disorder, Anxiety NOS, or Posttraumatic Stress Disorder).

The same pattern of disorders continued at follow-up, though at a reduced prevalence rate compared to baseline. Half of participants did not meet criteria for a DSM-IV diagnosis at time of follow-up (*n* = 53, 52%). Mood disorders were the most prevalent disorder at follow-up, with 33% of the sample having experienced mood disorder within the last month (*n* = 34) (figure 3). However, rates of mood disorder at follow-up were substantially lower than at baseline. There was a similar reduction in prevalence of anxiety disorders, with one-quarter of participants experiencing a current disorder at follow-up (*n* = 26). Substance use disorder rates remained consistent at baseline and follow-up, with 13% (*n* = 13) of participants experiencing an SUD within a month of follow-up assessment. There was no relationship between time to follow-up and mental disorder at follow-up (χ^2^(1, *n* = 102) = 0.211, *P* = .65, phi = 0.05). There were no differences in likelihood of diagnosis at baseline between gender for any of the disorders [mood χ^2^ (1, *n* = 100) = 1.724, *P* = .19, phi = 0.13; anxiety χ^2^ (1, *n* = 100) = 0.017, *P* = .90, phi = 0.13; SUD χ^2^ (1, *n = *100) = 0.007, *P* = .10, phi = 0.01]. Age at follow-up made no difference to the likelihood of having a disorder at follow-up [mood disorder, *t*(100, *n* = 102) = 0.533, *P* = .595; anxiety disorder, *t*(100, *n* = 102) = 0.311, *P* = .77; or SUD *t*(100, *n* = 102) = 0.310, *P* = .53, at follow-up]. However, males were significantly more likely to have a SUD at follow-up (23.1%) than females (6.3%), χ^2^ (1, *n* = 102) = 6.061, *P* = .014, phi = 0.24. There were no significant gender differences for other disorders [mood disorder, χ^2^ (1, *n* = 102) = 0.187, *P* = .67, phi *=* 0.04; anxiety disorder χ^2^ + (1, *n* = 102) = 3.395, *P* = .07, phi = 0.18].

### Course and Stability of Baseline Non-psychotic Mental Disorders

Over the follow-up period, only 6% of participants (*n* = 6) had not received a diagnosis of any DSM-IV disorder at either baseline or the follow-up assessment. Thirty-five percent of participants had *incident* non-psychotic disorders over the follow-up period (*n* = 35). Forty-four percent of participants remitted from their disorders (*n* = 54), while over half experienced their disorders at baseline and follow-up (“continuous” disorder, *n* = 54, 54%).

**Fig. 4.**
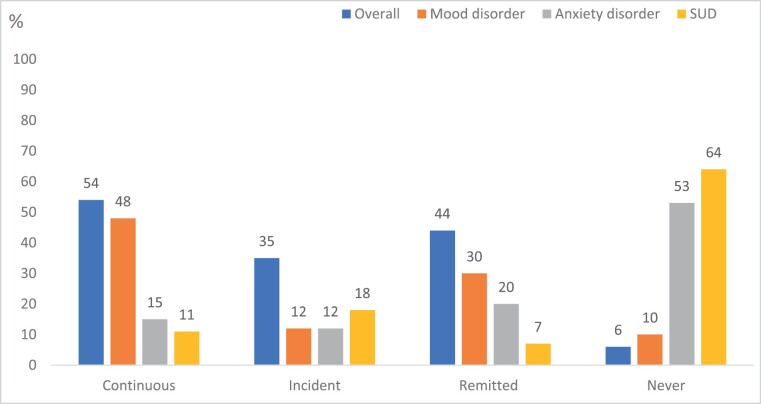
Trajectory of disorder—overall and by type of disorder. *Trajectory percentages for overall do not sum to 100% as some participants had more than one type of mental disorder.

**Fig. 5.**
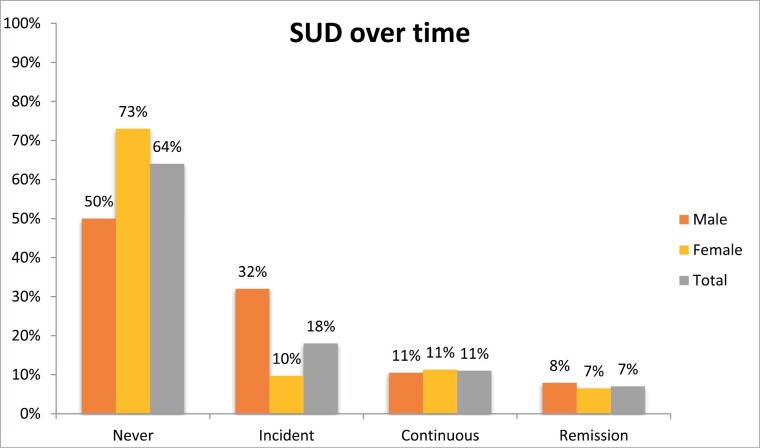
Trajectory of SUD by gender.

The disorder that was the most persistent over time was mood disorder (*n* = 48, 48%) (figure 4). Given the high prevalence of mood disorders at baseline, this disorder also had the highest rates of remittance (*n* = 30, 30%). Males were significantly more likely (*n* = 12, 32%) to have an incident SUD than females (figure 5) (*n* = 6, 10%), χ^2^ (3, *n* = 100) = 8.238, *P* = .04, phi = 0.21.

Age at follow-up was not associated with outcomes at follow-up for mood disorder (*t*(100, *n* = 102) = 0.533, *P* = .595; anxiety disorder, *t*(100, *n* = 102) = 0.311, *P* = .77; or SUD, *t*(100, *n* = 102) = 0.310, *P* = .53, or any disorder overall at follow-up, *t*(100, *n* = 102) = 1.091, *P* = 0.193).

Multinomial regression revealed that time to follow-up was not associated with course of mood disorder, (χ^2^(294) = 236.86, *P* = .994, phi = 0.05; anxiety disorder χ^2^(294) = 236.70, *P* = .994).

SUD over time (χ^2^(294) = 201.80, *P* = 1). Multinomial regression of age at baseline had no effect on the course of any disorder (Supplementary table 1).

### Persistence of UHR Status

Data were available for 84 participants on APS threshold at both time points. Of these participants, 29% (*n* = 24) had APS at a threshold (ie, symptom severity and frequency) that fulfilled UHR criteria at follow-up. Seventy-one percent of participants had remitted from UHR status by follow-up (*n* = 60).

A chi-square test for independence showed a significant association between mental disorders at follow-up (current) and UHR status at follow-up, χ^2^(1, *n* = 84) = 7.76, *P* < .01, phi = 0.30, with a greater proportion of individuals who still met UHR criteria reporting presence of a mental disorder within the last month (Supplementary table 2).

### Disorder Trajectory and Functional Outcomes at Follow-up

A one-way between-groups analysis of variance was conducted to explore the impact of trajectory of disorder groups on levels of functioning (SOFAS) at follow-up. There was a statistically significant difference in SOFAS scores for the four mood disorder trajectory groups, *F*(3,98) = 6.29, *P* < .001, η^2^ = 0.17, representing a small effect. Post hoc comparisons using the Tukey HSD test indicated that the mean SOFAS score for those with a continuous mood disorder (*M* = 65.96, *SD* = 10.32) was significantly worse than the mean SOFAS score of those with a remitted mood disorder (*M* = 75, *SD* = 9.60). The mean SOFAS score for those who had an incident mood disorder was not statistically different from the other groups (*M* = 69.33*, SD* = 7.62). The mean SOFAS score for those who never had a mood disorder was similar to those who had remitted (*M* = 74.40, *SD* = 6.39) and was not statistically different to those who had a continuous or incident mood disorder.

Trajectory of SUD over time was not related to level of functioning at follow-up: *F*(3, 98) = 2.35, *P* = .08 (Supplementary table 3). The trajectory of anxiety disorders was also not related to level of functioning at follow-up did: *F*(3, 118) = 2.48 *P* = .07 (Supplementary table 4).

## Discussion

There were three main areas of interest in this study: the trajectory of non-psychotic mental disorders in UHR individuals; persistence of UHR status over the long-term; and long-term functional outcomes in UHR individuals. In particular, the course of non-psychotic disorders in UHR participants was used to examine whether UHR is a specific risk state for psychosis outcomes or for a range of diagnostic outcomes. The study demonstrated: (a) that non-psychotic mental disorders were highly prevalent in UHR individuals at clinic entry and over the longer-term; (b) there were high rates of continuous mental disorders at follow-up and (c) high incidence of new mental disorders; (d) UHR status persisted in just under a third of the sample; and (e) trajectory of mental disorder was associated with functional outcomes over the long-term, with continuous mood disorder being associated with the poorest functional outcomes.

Only 13% of participants did not have a non-psychotic mental disorder at baseline. The proportion of participants without a mental disorder increased at follow-up, with almost half of participants (48.8%) not meeting diagnostic criteria at follow-up. Mood disorders remained the most prevalent, followed by anxiety disorders and SUDs at both time points. These results are consistent with previous findings.^9,15,26^ Rates of mental disorders were higher at follow-up than would be expected in the general population of people of a similar age.^35,36^

Looking at the trajectories of these disorders over time, only 6% of the sample never met criteria for a non-psychotic mental disorder. The most common experience for participants was to have a continuous non-psychotic mental disorder (52%); of those who entered the clinic with a mental disorder, a substantial proportion continued to experience clinically significant symptoms, with over a third developing an incident disorder (41.6%). A similar proportion of those entering the PACE clinic at baseline with a non-psychotic mental disorder (43.2%) would go on to remit from their disorder over time. This demonstrates that while there were positive outcomes for a reasonable proportion of participants, many were still experiencing significant non-psychotic mental health problems at follow-up.

The current study’s trajectory rates are mostly consistent with previous findings reported by Lin et al,^14^ with the exception of remitted disorders, Lin et al, finding a rate of 26% remitted disorders compared to the current study’s higher rate of 43.2%. However, the Lin et al^14^ follow-up covered a shorter period (mean of 6.9 years, with some substantially shorter follow-up times), whereas the current study’s follow-up averaged 8.8 years, with a minimum of 6 years, perhaps allowing more time for participants to remit from symptomatology present at baseline. Another point of difference is that while the Lin et al^14^ study found that female gender was a significant predictor of persistent or recurrent mood disorder compared to remitted mood disorder, and having incident anxiety disorder compared to never having an anxiety disorder, the current study did not find this. Both the Lin et al^14^ study and the current study had overrepresentation of females in the sample, which Lin et al^14^ proposed could have explained their higher rates of mood and anxiety disorders. However, despite the current study having the same overrepresentation, we instead found that male gender was associated with incidence of a SUD (32%, compared to 10% of females), and higher rates of having SUD at follow-up. This pattern is similar with young males being twice as likely to have an SUD than females, though the incidence for both is greater in our sample than the general population (4.4% of males, 2.1% of females in general population).^37^ It could be that the longer follow-up time of the current study allowed for these disorders to develop. This highlights the importance of replication studies and extended up follow-up periods for obtaining outcome data.

The current study revealed that 29% of the sample still met UHR criteria at follow-up, consistent with the rate reported by Lin et al.^14^ Persistence of APS in these participants could be reflective of an ongoing risk for psychosis, given that the prodromal period has been known to last up to at least 10 years.^34^ Research supporting this idea comes from studies comparing help-seeking non-UHR individuals to UHR individuals. One such study revealed psychosis to be the only incident disorder that differed significantly between the two groups,^38^ with another comparison study between non-UHR and UHR help-seekers also supporting the notion that APS meeting UHR threshold were specific to predicting psychosis.^39^

In contrast, it is also possible that APS are a feature of severe non-psychotic disorders, as indicated in studies linking sub-threshold psychotic symptoms to concurrent depressive, anxiety, and phobic symptoms.^19,40–42^ The current study did indeed find an association between APS and non-psychotic mental disorders, which supports this possibility, but does not, however, preclude the possibility of a long psychosis prodrome in these individuals. Further long-term outcome studies are required to clarify the clinical trajectories of individuals with persistent APS.

The current study explored the association between trajectory of mental disorder (mood, anxiety, and SUD) and functional outcomes over the longest time-frame thus far examined in non-transitioned UHR participants. As predicted, participants with a continuous mental disorder had the worst functional outcomes as measured by SOFAS, consistent with findings by Rutigliano et al.^19^ Functional outcomes were best for those who had remitted, with these participants were doing significantly better than those who with a continuous disorder. Participants with an incident disorder had lower functioning than the remitted and never groups, but higher than participants with a continuous disorder. Similar patterns were observed for specific disorder groups, with some variation. Comorbid mental disorders have previously been shown to be associated with lower functioning in UHR participants at baseline.^26^ These results suggest that UHR participants are experiencing mental health problems that are disabling and distressing in their own right, irrespective of whether or not the individual progresses to frank psychosis.

It was common for even those without a non-psychotic disorder at baseline to develop a non-psychotic disorder over time. These findings are in line with Beck et al’s^9^ observation that over the long-term, UHR individuals do not achieve full clinical remission. Beck et al^9^ systematic review noted a lack of long-term trajectory data in the relevant literature, concluding that further research is required.

While the UHR criteria are associated with a 25% risk of developing psychosis within three years,^43^ the current report shows, consistent with previous findings,^44^ that they are also useful for identifying those at risk of long-term non-psychotic disorders. There is a need to better understand the risk factors for the persistence and incidence of non-psychotic disorders in the UHR population, in addition to those associated with risk for developing psychotic disorder. Sustained clinical attention to monitor and avert these outcomes is warranted. In this sense, UHR identification can serve a dual function of detecting psychosis risk and heightened risk of transdiagnostic outcomes (which are not mutually exclusive), consistent with previous findings in general youth mental health services that the UHR phenotype is a poor prognostic marker transdiagnostically.^45^ Pluripotent risk criteria,^46,47^ expanding on the UHR criteria, have been introduced in recent years in an attempt to further maximize the identification of young people at transdiagnostic risk.

### Strengths and Limitations

The strengths of this study include a long-term follow-up (minimum of 6 years), with high follow-up rates, and a relatively large sample from one site. It is also a more homogenous sample from the larger sample from which it derives,^14^ given that the sample was recruited over a shorter time-frame, resulting in minimal changes in referral pathways and treatment exposure. A limitation was that follow-up diagnoses for 25 participants were established via a telephone-based assessment, which is not optimal for diagnostic assessment. The variation in time to follow-up from baseline (ranging from 6.8 to 12.1 years) introduces variability in the assessment of clinical trajectory and should be considered in interpretation.

### Conclusions

This study adds to the evidence base that a substantial proportion of UHR individuals experience persistent APS and persistent and incident non-psychotic disorders over the long term.^14,15^ While the UHR criteria are effective in identifying young people at risk of psychosis, they also pick up on transdiagnostic risk. Poorer functional outcomes in this sample were associated with continuous mood disorder. Because this UHR sample never developed a full threshold psychosis, they would not have been targeted for prolonged intervention. However, given their long-term impairment, this group would benefit from longer-term treatment both for their non-psychotic disorders and continuing APS. These findings indicate a need for long-term monitoring of non-transitioned individuals, as well as the option for re-entry into services if required.

## Supplementary Material

Supplementary material is available at https://academic.oup.com/schizophreniabulletin/.

sbae005_suppl_Supplementary_Material
