# Supplementary material for: Non-psychotic Outcomes in Young People at Ultra-High Risk of Developing a Psychotic Disorder: A Long-Term Follow-up Study
Source: Schizophr Bull. 2024 Feb 16;50(6):1279–86. doi: 10.1093/schbul/sbae005 (PMC11548927; doi:10.1093/schbul/sbae005)
Supplement: sbae005_suppl_Supplementary_Material [file sbae005_suppl_supplementary_material.docx]

Supplementary Table 1

*Multinomial regression results for association between age at baseline and meeting criteria for a mental disorder at follow-up*

| Age at baseline predicting disorder type at follow-up | B | Se(B) | Wald | Df | p-value | Odds Ratio (OR) | 95.0% CI for OR | |
| --- | --- | --- | --- | --- | --- | --- | --- | --- |
|  |  |  |  |  |  |  | Lower | Upper |
| Mood | -.005 | .074 | .005 | 1 | .941 | .995 | .860 | 1.150 |
| Anxiety | .001 | .080 | .000 | 1 | .989 | 1.001 | .856 | .171 |
| SUD | .054 | .101 | .282 | 1 | .596 | 1.055 | .865 | 1.287 |

Supplementary Table 2

*Relationship between presence of a non-psychotic mental disorder at follow up and meeting UHR criteria at follow- up*

|  |  | Met UHR criteria at follow-up | |  |
| --- | --- | --- | --- | --- |
|  |  | No | Yes | Total |
| Had a non-psychotic mental disorder at follow-up | No | 34 (55.7%) | 5 (21.7%) | 39 |
|  | Yes | 27 (44.3%) | 18 (78.3%) | 45 |
| Total |  | 61 | 23 | 84 |

Supplementary Table 3:

*Follow-up SOFAS scores and SUD trajectory: means and standard deviations*

|  | *M* | *SD* |
| --- | --- | --- |
| Incident SUD | 65.67 | 10.05 |
| Never had an SUD | 71.76 | 9.79 |
| Continuous SUD | 65.91 | 12.81 |
| Remitted from SUD | 70.00 | 7.64 |

Supplementary Table 4:

*Follow-up SOFAS scores and anxiety trajectory: means and standard deviations*

|  | *M* | *SD* |
| --- | --- | --- |
| Incident anxiety disorder | 64.50 | 11.70 |
| Never had an anxiety disorder | 71.13 | 10 |
| Continuous anxiety disorder | 66.67 | 10.29 |
| Remitted from anxiety disorder | 72.25 | 9.10 |
